# Gonadotropin-releasing hormone antagonist versus progestin for the prevention of premature luteinising hormone surges in poor responders undergoing in vitro fertilisation treatment: study protocol for a randomised controlled trial

**DOI:** 10.1186/s13063-018-2850-x

**Published:** 2018-08-22

**Authors:** Yun Wang, Yanping Kuang, Qiuju Chen, Renfei Cai

**Affiliations:** Department of Assisted Reproduction, Shanghai Ninth People’s Hospital, Shanghai Jiaotong University School of Medicine, Zhizaoju road no 639, Shanghai, People’s Republic of China

**Keywords:** GnRH antagonist, Progestin, Poor responders, LH surge

## Abstract

**Background:**

Progress in vitrification techniques has allowed reproductive physicians to consider new strategies for using progestin as an alternative to a GnRH analogue to improve in vitro fertilisation (IVF). However, the role of progestin in blocking luteinising hormone (LH) surges and its potential in clinical practice are unclear, especially for poor responders. We designed a prospective randomised controlled trial (RCT) to compare the efficacy of a gonadotropin-releasing hormone (GnRH) antagonist and progestin in blocking LH surges and premature ovulation in poor responders.

**Methods/design:**

Poor responders who meet the Bologna criteria will be randomised to one of two stimulation regimens—gonadotropin-releasing hormone (GnRH) antagonist or progestin-primed ovarian stimulation (PPOS)—using a computer-generated random number. Fresh embryos were transferred in the GnRH antagonist group and frozen embryos were transferred in the PPOS group. The primary outcome is the incidence of premature LH surges. Secondary outcomes include the number of oocytes retrieved, the number of embryos available for transfer, implantation rates and clinical pregnancy. The sample size for this trial is estimated as 340 participants, with 170 participants in each group. The data analysis will be by intention to treat.

**Discussion:**

To our knowledge, this is the first RCT to examine the efficacy of administering progestin orally to block LH surges and premature ovulation compared with the GnRH antagonist protocols in poor responders undergoing IVF treatment.

**Trial registration:**

www.chictr.org.cn. ChiCTR-IPR-17010906. Registered on 18 March 2017.

**Electronic supplementary material:**

The online version of this article (10.1186/s13063-018-2850-x) contains supplementary material, which is available to authorized users.

## Background

Gonadotropin-releasing hormone (GnRH) antagonists have been used to suppress pituitary activity and to prevent premature surges of luteinising hormone (LH) during controlled ovarian stimulation since the 1990s [1]. GnRH antagonist therapy does not produce a flare effect and rapidly suppresses gonadotropins and it can be initiated in the late follicular phase of the menstrual cycle. Thus, GnRH antagonists are considered to be beneficial for poor responders since there is less suppression in the early follicular phase [2]. Previous studies of poor responders have shown that a GnRH antagonist protocol is associated with decreased cycle cancellation and fewer days of gonadotropin stimulation, but the clinical pregnancy outcomes were not significantly different between GnRH antagonist and agonist protocols [3].

Progress in vitrification techniques has allowed reproductive physicians to consider new strategies for using progestin as an alternative to a GnRH analogue for improving in vitro fertilisation (IVF) [4–8]. Progestin can inhibit the pre-ovulatory LH surge when it is administered during the early part of the cycle before oestrogen priming [9–11]. Progestin also alters pituitary responsiveness to GnRH and gonadotrophin secretion [12, 13]. For more than 50 years, progestin has been widely applied to control ovulation in hormonal contraception [14], and since 2014, its use has been extended to preventing premature ovulation in IVF [5, 6]. Our recent studies demonstrated that progestin-primed ovarian stimulation (PPOS) produced a gradually decreasing LH level during ovarian stimulation, with a low incidence of LH surges (0.15%) in women with a normal ovarian reserve [6]. PPOS also produces an acceptable pregnancy outcome compared with the conventional short protocol [4–8]. However, currently, there are no data comparing the efficacy and safety of a GnRH antagonist and progestin in blocking LH surges and premature ovulation in poor responders.

GnRH antagonists have a reported failure rate of approximately 0.34–8.0% for controlling premature LH surges in women with a normal ovarian reserve [1–3]. An antagonist failure is more likely to occur in women with advanced age, a diminished ovarian reserve and poor response to gonadotropins [15]. Our prospective trial in a population of poor responders showed that ovulation of the dominant follicle was controlled well using progestin administered orally as a surrogate for a GnRH antagonist, with low incidence rates of LH surges and premature ovulation (3.0%) [8]. Moreover, progestin priming prolonged the follicular phase by one more day, and the diameter of the pre-ovulatory follicle was larger than those of natural cycle patients. These data indicated that progestin treatment significantly suppressed follicular rupture and provided a wide window for oocyte retrieval [8]. Therefore, we assumed that progestin may show some superiority in controlling premature LH surges compared with GnRH antagonists in poor responders. Thus, we have developed this well-designed large-sample prospective trial to investigate the potential of progestin for poor responders undergoing IVF treatment.

## Methods/design

In this trial, the efficacies of a GnRH antagonist and progestin are being compared in 340 poor responders undergoing IVF through intracytoplasmic sperm injection (ICSI). The participants will be enrolled in Shanghai Ninth People’s Hospital affiliated with Shanghai Jiaotong University School of Medicine. The study has been approved by the Institutional Review Board of Shanghai Ninth People’s Hospital (2016–198-T142). Informed consent will be obtained from each patient before any study procedure is performed, in accordance with good clinical practice.

**Fig. 1:**
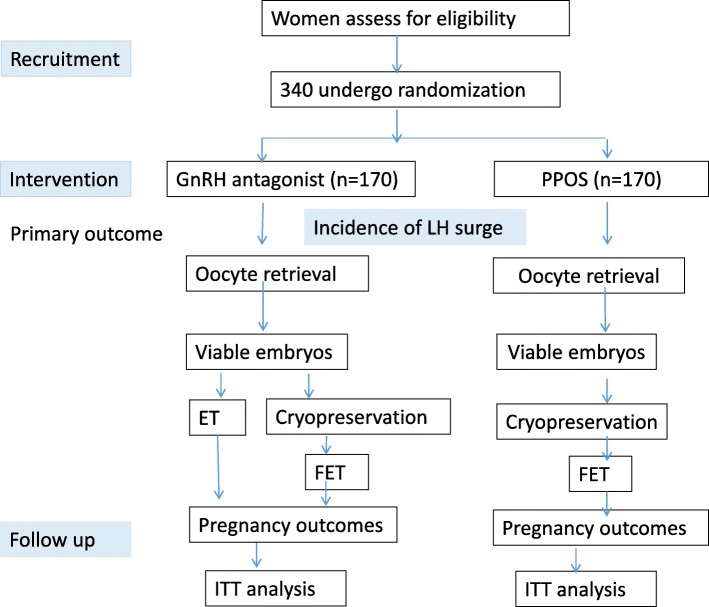
Flowchart of this randomised controlled trial comparing PPOS with a GnRH antagonist in poor responders. ET embryo transfer, FET frozen-thawed embryo transfer, GnRH gonadotrophin-releasing hormone, ITT intention to treat, LH luteinising hormone, PPOS progestin-primed ovarian stimulation

This protocol has been written in accordance with the Standard Protocol Items: Recommendations for Interventional Trials (SPIRIT). A SPIRIT checklist is provided in Additional file 1. Any significant modifications to the protocol will require a formal protocol amendment, agreed by the project team and approved by our Institutional Review Board. Minor administrative changes to the protocol will be documented in a memorandum. The study flowchart is shown in Fig. 1.

### Participants

#### Inclusion criteria

The following are the inclusion criteria:

1. Women who have a history of infertility ≥1 year
2. Women aged >22 and <42 years
3. Women with spontaneous menstrual cycles of 21–35 days
4. Women who have at least one of the following indications for IVF or ICSI: tubal factor, male factor, diminished ovarian reserve, endometriosis or unexplained factors
5. Women diagnosed as poor responders according to the Bologna criteria, including at least two of the three following criteria:
  - Advanced age (≥40 years) or any other risk factor for poor ovarian response
  - A previous poor response with no more than three oocytes retrieved using the conventional stimulation protocols
  - Abnormal ovarian reserve test results, including bilateral antral follicle counts <7 or serum anti-Müllerian hormone <1.1 ng/ml

#### Exclusion criteria

Women who met any of the following criteria are excluded:

1. Clinically significant systemic diseases, such as renal failure and systemic lupus erythematosus
2. Premature ovarian insufficiency
3. Up to five previous unsuccessful IVF attempts
4. Known Müllerian anomalies
5. Any contraindications to ovarian stimulation treatments
6. Unable to comply with the study procedures

### Randomisation

Participants will be allocated randomly into one of the two arms at a ratio of 1:1 on menstrual cycle day 3. The allocation sequence will be generated utilising computer-generated random numbers. Both investigators and participants will be aware of the allocation after ovarian stimulation. The doctors and embryologists involved in oocyte retrieval and embryo transfer are blinded to the group assignments of the participants in the trial.

### Protocols

#### GnRH antagonist protocol

The flexible GnRH antagonist protocol is as follows: 150–225 IU of human menopausal gonadotropin (hMG) is administered daily from menstrual cycle day 3. After 5 days of injections, when the dominant follicles reach a diameter of approximately 14 mm, 0.125–0.25 mg of GnRH antagonist is administered daily up to the trigger day. For women with a low or normal body mass index (<25.0 kg/m^2^) or low LH levels before GnRH antagonist administration (<2.0 mIU/ml), 0.125 mg of antagonist is administered daily. For women with a higher body mass index (≥25.0 kg/m^2^) or LH levels ≥2.0 mIU/ml, 0.25 mg antagonist will be used daily up to the trigger day. The dose of hMG is adjusted according to the ovarian response. When the dominant follicles reach a diameter of 18 mm, the final stage of oocyte maturation is induced with 100 μg of triptorelin via subcutaneous injection and 5000 IU of human chorionic gonadotrophin (hCG) via intramuscular injection. Oocyte retrieval is performed 36 h later.

#### PPOS protocol

hMG at 150–225 IU and medroxyprogesterone acetate (MPA) at 10 mg are administered daily from cycle day 3. Five days later, the hMG dose is adjusted according to the ovarian response, while the MPA dose is kept the same up to the trigger day. When the dominant follicles reach a diameter of 18 mm, the final stage of oocyte maturation is induced with 100 μg of triptorelin via subcutaneous injection and 5000 IU of hCG via intramuscular injection. Oocyte retrieval is performed 36 h later.

### In vitro fertilisation and embryo culture

All follicles more than 10 mm will be retrieved. Follicles are flushed three times at most if no cumulus oocyte complex is present. Standard insemination or ICSI is performed within 6 h of retrieval. Embryos are examined for the number and regularity of blastomeres and the degree of embryonic fragmentation on the third day. If available, two top-quality embryos (including grade I and grade II, eight-cell blastomere embryos) in the GnRH antagonist group are transferred on the third day. The remaining top-quality embryos are frozen by vitrification, while the non-top-quality embryos are cultured for an extended time. Only blastocysts with good morphology are frozen on day 5 or 6. All top-quality cleavage embryos and the cryopreserved blastocysts are recorded as viable embryos. In the PPOS group, all top-quality embryos are frozen on the third day, while the non-top-quality embryos are cultured for an extended time and cryopreserved according to the same criteria as above.

### Endometrium preparation and frozen-thawed embryo transfer

Endometrium preparation uses either mild stimulation or hormone replacement therapy. For mild stimulation frozen-thawed embryo transfer cycles, we prescribe letrozole 2.5–5 mg for 3–5 days from cycle day 3 and then monitor follicular growth via serum hormone levels and ultrasound from cycle day 10. At times, treatment includes a low dose of hMG (75 IU/day) to stimulate follicle growth and the endometrial lining. When the diameter of the dominant follicle is >16 mm and the endometrial thickness is > 8 mm, with *E*_2_ > 150 pg/ml and progesterone <1.0 ng/ml, one of two procedures is performed, depending upon the LH level. If the serum LH level is <20 mIU/ml, 5000 IU of hCG is administered at night (21:00) to trigger ovulation, and the transfer of cleavage embryos is arranged for 5 days later. If the LH level is >20 mIU/ml, 5000 IU of hCG is injected the same afternoon, and the transfer is conducted 4 days later. The blastocyst transfer is arranged on the sixth or seventh day depending on serum hormone levels and ultrasound results. Dydrogesterone (Abbott Biologicals BV, the Netherlands) was administered orally at 40 mg/day and micronised progesterone capsules at 400 mg/day was administered vaginally for luteal support beginning on the third day after hCG injection.

For patients with a thin endometrium or where the frozen-thawed embryo transfer fails after stimulation cycles, hormone replacement therapy is recommended for endometrial preparation, specifically ethinyl oestradiol administered orally at 75 mcg/day from cycle day 3 onwards, which is commonly used for 14 days. Once the endometrial lining is >8 mm thick, Femoston (a yellow tablet) is administered twice per day (Abbott Healthcare Products BV, Weesp, the Netherlands), and 400 mg micronised progesterone capsules are administered daily via an intravaginal route. The time for thawing and transfer is determined on the third day after progesterone administration. The maximum number of transferred embryos is two per cycle. When pregnancy is achieved, the progesterone supplement is continued until 10 weeks of gestation.

### Hormonal measurement

Serum follicle-stimulating hormone (FSH), LH, oestradiol and progesterone levels are monitored during the ovarian stimulation. Hormone levels are measured via chemiluminescence (Abbott Biologicals BV, The Netherlands).

### Outcome measurements

#### Primary outcome

The primary outcome is the incidence of premature LH surges, defined as the serum LH > 15 mIU/ml on the trigger day, with or without dominant follicle rupture and increased serum progesterone. Premature ovulation is defined as dominant follicle rupture before the scheduled time. Increased progesterone alone is not defined as the presentation of an LH surge and is listed independently.

#### Secondary outcomes

Secondary efficacy parameters include the number of oocytes retrieved, the number of viable embryos, the clinical pregnancy rate, the implantation rate, the ongoing pregnancy rate and the cumulative live birth rates from a single IVF cycle. Clinical pregnancy will be defined as the presence of an intrauterine gestation sac at 7 weeks of gestation. Ongoing pregnancy will be defined as a viable pregnancy at 12 weeks of gestation.

The safety endpoints include the incidence rates of ovarian hyperstimulation syndrome, miscarriage, ectopic pregnancy, pregnancy complication, congenital anomalies and neonatal complications.

### Statistics

#### Sample size and power calculations

For the power calculation, previous studies reported that the incidence of premature LH surges in the GnRH antagonist protocol was 8.0%, and our recent data show that the incidence of premature LH surges and ovulation was 3.0% in poor responders using the PPOS protocol. Therefore, we hypothesise that the administration of MPA would decrease the incidence of premature LH surges. The superiority margin is 4.0%. A sample size of 166 in each group would yield 90% power to establish superiority at the 0.01 level of significance, and 109 in each group yield 90% power to establish superiority at 0.05 level of significance [16]. Given the abundant clinical resources in our clinic, the number of participants is set as 170 in each group in this trial.

### Data management

**Fig. 2:**
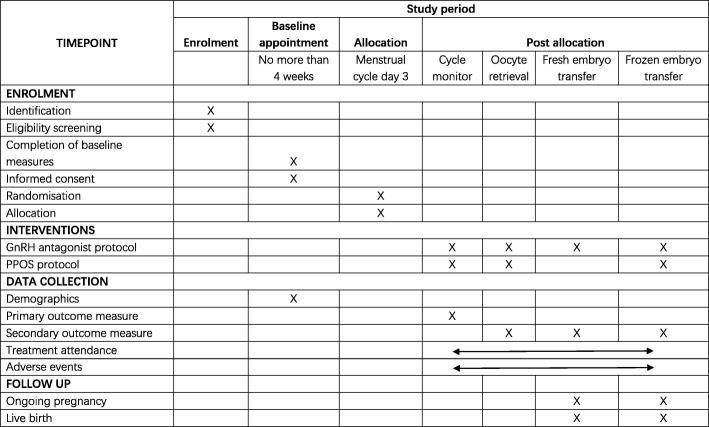
SPIRIT diagram for this protocol comparing PPOS with GnRH antagonist in poor responders. GnRH gonadotrophin-releasing hormone, PPOS progestin-primed ovarian stimulation

The timepoints of enrolment, intervention, data collection and follow-up are described in Fig. 2. Data collected are entered into our electronic data capture system and stored on a secure server at Shanghai Ninth People’s Hospital. An automated system for validating data against a set of predefined rules will query investigators regarding data that are invalid, illogical or incomplete. Data elements critical to the primary aim of this trial are double-checked to confirm the accuracy of the data entered compared with the source documents.

### Statistical analysis

We will utilise an intention-to-treat approach with a chi-square test to examine differences in the incidence of premature LH surges. Secondary efficacy parameters and safety parameters will be analysed using a chi-square test for enumeration data and Student’s *t* test for measurement data. *p* < 0.05 is considered as a significant difference.

## Discussion

How to control premature LH surges in poor responders has long been an issue in IVF treatment. These poor responders have small quantities of primordial follicle pools and FSH-sensitive follicles, wherein the follicles biologically mature quickly and are prone to premature luteinisation [17]. Therefore, it is more difficult to control premature LH surges in poor responders than in those with a normal ovarian reserve. GnRH antagonists accomplish pituitary suppression via a competitive blockage of the GnRH receptor, but the capability of the endogenous oestrogen-induced GnRH release is still preserved, and a small proportion of antagonist cycles fail to control LH surges, especially in those of advanced age and with a diminished ovarian reserve [18–20]. In preliminary studies, progestin has been shown to inhibit premature ovulation effectively, and it is useful to compare antagonists and progestin in controlling premature ovulation in poor responders.

To our knowledge, this is the first randomised controlled trial to examine the efficacy of progestin administered orally in blocking LH surges and premature ovulation during ovarian stimulation for poor responders compared with the standard GnRH antagonist protocols. The study results will add to current knowledge on controlled ovarian stimulation and will have the potential to establish a better treatment for poor responders.

### Trial status

The study was conceived and designed in 2016. The registry number is ChiCTR-IPR-17010906 and it was registered on 18 March 2017 (http://www.chictr.org.cn/showproj.aspx?proj=11024). The first participant was randomised on 20 March 2017. We will complete recruitment in July 2018, and patient follow-ups will be ongoing. This protocol, version 2, was approved on 12 January 2017.

## Additional file

Additional file 1: SPIRIT checklist. (DOC 100 kb)

## Abbreviations

ET: Embryo transfer
FET: Frozen-thawed embryo transfer
FSH: Follicle-stimulating hormone
GnRH: Gonadotrophin-releasing hormone
hCG: Human chorionic gonadotrophin
hMG: Human menopausal gonadotropin
ICSI: Intracytoplasmic sperm injection
ITT: Intention to treat
IVF: In vitro fertilisation
LH: Luteinising hormone
MPA: Medroxyprogesterone acetate
PPOS: Progestin-primed ovarian stimulation
